# Radiomic analysis of the peritumoral zone identifies imaging signatures of glioma invasion associated with HSP70 expression

**DOI:** 10.3389/fonc.2026.1881576

**Published:** 2026-06-12

**Authors:** Natalia Mikhailova, Anastasiia Nechaeva, Danila Bobkov, Hem Chandra Jha, Kadambari KV, Yerramneni Vamsi Krishna, Hari Ponnamma Rani, Aleksander Kim, Maxim Shevtsov

**Affiliations:** 1 Polenov Russian Research Neurosurgical Institute, Almazov National Medical Research Centre, St. Petersburg, Russia; 2 Laboratory of Biomedical Nanotechnologies, Institute of Cytology of the Russian Academy of Sciences (RAS), St. Petersburg, Russia; 3 Department of Biosciences and Biomedical Engineering, IIT Indore, Indore, India; 4 Department Of Computer Science & Engineering, National Institute of Technology Warangal (NITW), Warangal, Telangana, India; 5 Nizam’s Institute of Medical Sciences, Hyderabad, India; 6 Department of Mathematics, National Institute of Technology Warangal (NITW), Warangal, Telangana, India; 7 Department of Radiation Oncology, Klinikum rechts der Isar, Technical University of Munich, Munich, Germany

**Keywords:** glioma, Hsp70, imaging biomarkers, magnetic resonance imaging, peritumoral region, radiomics, tumor invasion, tumor microenvironment

## Abstract

**Objective:**

Glioma invasion into surrounding brain tissue drives disease progression but is challenging to evaluate with conventional MRI. This study aimed to determine whether radiomic features extracted from the peritumoral zone capture biologically relevant invasion-associated imaging phenotypes, using membrane-bound Hsp70 (mHsp70) as a molecular correlate.

**Methods:**

A retrospective study was performed using preoperative MRI data from 80 patients with glioma, including adult glioblastoma (GBM), adult lower-grade glioma (LGG), and pediatric GBM/ATRT cases. Voxel-wise radiomic features were extracted from the peritumoral region and used to train a stochastic gradient descent (SGD) classifier to distinguish GBM-associated invasion-enriched imaging phenotypes from LGG-associated comparison phenotypes. Patient-level cohort separation was used throughout model development to prevent data leakage. Feature importance was assessed using SHAP analysis. In a biologically validated subgroup of 23 patients, membrane-bound Hsp70 (mHSP70) expression was quantified in peritumoral tissue samples using confocal microscopy. Patient-level radiomic invasion metrics were correlated with mHsp70 fluorescence intensity.

**Results:**

The SVM classifier demonstrated good discriminative performance, achieving an AUC of 0.875 (95% CI: 0.736–0.896) in the independent test cohort. SHAP analysis identified higher-order texture features, including GLCM-, GLRLM-, and LoG-derived radiomic descriptors, as major contributors to model predictions. Reproducibility analysis demonstrated good-to-excellent agreement for all 20 SHAP-selected features, with mean ICC values ranging from 0.859 to 0.986. In the biological validation subgroup, the radiomics-derived Invasion Burden Index (IBI) showed a significant positive correlation with mHsp70 expression (Spearman ρ = 0.67, p = 0.0004, 95% CI: 0.232–0.805, n = 23). Leave-one-out sensitivity analysis demonstrated stable correlations, indicating that the observed association was not driven by individual cases. Regions exhibiting elevated invasion-associated radiomic signatures frequently extended beyond the conventional MRI-defined tumor margin and showed spatial correspondence with areas of subsequent tumor progression on follow-up imaging.

**Conclusions:**

Voxel-wise radiomic analysis of the peritumoral zone identifies invasion-associated imaging phenotypes that correlate with membrane Hsp70 expression, a biological marker associated with aggressive tumor behavior. These findings support the potential utility of radiomic invasion mapping as a non-invasive tool for characterizing infiltrative glioma biology and generating hypotheses regarding patterns of tumor progression. Further prospective studies with spatially matched biological validation are needed.

## Introduction

Radiomics is a quantitative approach to medical imaging that aims to extract clinically relevant information from biomedical images through advanced mathematical analysis. It is based on the premise that medical images contain information about underlying biological processes that is not accessible through conventional visual assessment (1). By quantifying spatial patterns of signal intensity, texture, and voxel relationships, radiomics enables objective characterization of tissue heterogeneity using computational and machine learning techniques (2).

A central concept in radiomics is the extraction of imaging biomarkers, which are quantitative features derived from modalities such as MRI, CT, PET, and ultrasound. These features have been proposed as non-invasive surrogates of tumor biology, often described as a form of “virtual biopsy.” Previous studies have demonstrated associations between radiomic features and cellular heterogeneity, as well as genomic, transcriptomic, and proteomic characteristics (3, 4). However, the robustness of radiomic analysis depends on factors such as image acquisition, preprocessing, and segmentation, which may influence feature stability and reproducibility.

Although many radiomic features are based on established texture analysis methods (5), the innovation of radiomics lies in the high-throughput extraction and integrative analysis of large numbers of features. This enables identification of complex patterns associated with disease progression, prognosis, and treatment response, particularly in oncology.

In gliomas, tumor invasion into the surrounding brain tissue is a key determinant of recurrence and clinical outcome (6–8). Conventional MRI is limited in its ability to distinguish infiltrative tumor from peritumoral edema, as both may appear similar on standard imaging sequences (9). As a result, the peritumoral region represents a critical but poorly characterized zone where invasive tumor cells may be present but remain undetected by visual assessment.

Several studies have applied radiomic approaches to characterize tumor infiltration and predict recurrence patterns. For example, Dasgupta et al. (10) utilized edema regions from patients with low-grade gliomas and brain metastases to model invasive behavior, while Akbari et al. (11) incorporated multiparametric MRI features, including diffusion tensor imaging and perfusion imaging, to assess peritumoral infiltration. However, these approaches often focus on spatial prediction of recurrence or rely on advanced imaging modalities that are not routinely available in clinical practice.

Invasion is a hallmark of glioma biology and is driven by a complex interplay of cellular, molecular, and microenvironmental factors (12). Glioma cells exhibit remarkable plasticity, enabling them to migrate along white matter tracts, blood vessels, and other structural pathways within the brain. This invasive capacity is associated with alterations in cell adhesion, extracellular matrix remodeling, cytoskeletal dynamics, and stress response pathways (13). Understanding these processes is essential for developing more effective therapeutic strategies and for improving the interpretation of imaging findings.

Among the molecular pathways implicated in tumor invasion, the heat shock protein (HSP) family has gained increasing attention (14). Heat shock proteins are highly conserved molecular chaperones that play critical roles in protein folding, stabilization, and cellular stress responses (15). Hsp70, in particular, is one of the most extensively studied members of this family and is known to be upregulated in a wide range of malignancies, including gliomas (16–18). Elevated Hsp70 expression has been associated with increased tumor cell survival, resistance to apoptosis, and enhanced adaptability to environmental stressors such as hypoxia and nutrient deprivation. Beyond its intracellular functions, Hsp70 can also be expressed on the cell surface as membrane-bound Hsp70 (mHsp70), a phenomenon that appears to be largely restricted to tumor cells (19–21). The presence of mHsp70 has been linked to aggressive tumor phenotypes and may play a role in modulating interactions between tumor cells and their microenvironment. In gliomas, mHsp70 expression has been associated with increased migratory and invasive behavior, potentially through its involvement in cytoskeletal organization, membrane stability, and signaling pathways that regulate cell motility, as was shown previously by our group (22, 23). Furthermore, mHsp70 may influence immune recognition and tumor-immune system interactions, adding another layer of complexity to its role in tumor biology (24).

The analysis of Hsp70, and specifically its membrane-bound form, is therefore of considerable interest in the context of glioma invasion. As a stress-inducible protein, Hsp70 reflects the cellular response to the hostile tumor microenvironment, including hypoxia, oxidative stress, and metabolic challenges. Its overexpression may confer a survival advantage to tumor cells at the invasive front, where conditions are particularly unfavorable. Additionally, mHsp70 may serve as a marker of cellular transformation and malignancy, distinguishing invasive tumor cells from normal brain tissue (22). These properties make Hsp70 an attractive candidate for investigating the biological basis of imaging-derived features and for identifying potential links between radiological patterns and molecular mechanisms.

In contrast to traditional imaging studies that focus primarily on the localization of recurrence-prone regions, the present study aims to identify radiomic signatures associated with fundamental biological mechanisms of glioma invasion using features extracted from conventional MRI. Rather than limiting the analysis to spatial patterns of tumor recurrence, we investigate whether radiomic features capture intrinsic characteristics of tumor aggressiveness and invasiveness at the population level. This approach reflects a shift from descriptive imaging toward a more integrative framework that seeks to uncover the biological underpinnings of radiological phenotypes.

A key aspect of this study is the validation of imaging-derived features through correlation with molecular data obtained from peritumoral tissue samples. Specifically, we employ confocal microscopy to quantify membrane-bound Hsp70 expression in regions adjacent to the tumor core. These peritumoral areas are of particular interest because they represent the interface between tumor and normal brain tissue and are critical sites of invasion. By focusing on mHsp70 expression in these regions, we aim to capture a molecular signature of invasive tumor behavior that can be directly compared with radiomic features extracted from MRI.

The integration of radiomic analysis with molecular measurements addresses a major challenge in the field: the biological interpretation of imaging features. While radiomics has demonstrated considerable potential in predicting clinical outcomes and molecular characteristics, the underlying biological meaning of many radiomic features remains unclear. Establishing direct links between imaging features and specific molecular processes is essential for advancing radiomics from a purely data-driven approach to a biologically informed discipline. In this context, the use of mHsp70 as a marker of invasion provides a mechanistically relevant target for validation.

Furthermore, the focus on conventional MRI sequences enhances the clinical applicability of the study. Advanced imaging modalities, such as diffusion tensor imaging or perfusion-weighted imaging, can provide additional information about tumor biology but are not always available in routine clinical practice. By demonstrating that meaningful radiomic signatures of invasion can be derived from widely used MRI sequences, this study supports the potential for broad implementation of radiomics in clinical workflows. This is particularly important in the management of glioma patients, where timely and accessible diagnostic tools are essential (25).

Another important consideration is the potential role of radiomic features as non-invasive biomarkers of tumor invasion (25, 26). Currently, the assessment of invasive behavior relies largely on histopathological analysis, which is limited by sampling bias and the inability to capture the full spatial extent of the tumor. Radiomics offers the possibility of evaluating the entire tumor and its surrounding environment in a non-invasive manner, providing a more comprehensive view of tumor heterogeneity and invasion. If validated, radiomic signatures associated with mHsp70 expression could serve as surrogate markers of invasive potential, aiding in treatment planning and risk stratification. In addition, the identification of imaging correlates of mHsp70 expression may have therapeutic implications. mHsp70 has been explored as a potential target for cancer therapy, and several strategies aimed at inhibiting its function or exploiting its immunogenic properties are under investigation (19, 27). If radiomic features can reliably reflect mHsp70 expression, they could be used to identify patients who are most likely to benefit from such therapies. This would further enhance the clinical relevance of radiomics and support its role in guiding treatment decisions.

The present study also contributes to the growing body of literature on the tumor microenvironment and its role in cancer progression (28, 29). The peritumoral region is increasingly recognized as a critical determinant of tumor behavior, influencing processes such as invasion, angiogenesis, and immune response. By focusing on this region and examining the relationship between imaging features and molecular markers of invasion, this study highlights the importance of considering the tumor in its broader biological context. This perspective may lead to new insights into the mechanisms of glioma progression and to the identification of novel therapeutic targets.

In summary, this study aims to advance the understanding of glioma invasion by integrating radiomic analysis of conventional MRI with molecular validation using membrane-bound mHsp70 expression in peritumoral tissue. By moving beyond the localization of recurrence and focusing on the biological processes underlying tumor invasion, we seek to establish a link between imaging phenotypes and molecular mechanisms. The analysis of Hsp70, particularly its membrane-bound form, provides a biologically meaningful marker of invasive behavior and offers a unique opportunity to interpret radiomic features in a mechanistic context. Through this integrative approach, the study explores the potential of radiomics to serve as a non-invasive surrogate of tumor invasion, ultimately contributing to improved characterization of glioma biology and enhancing the role of medical imaging in precision oncology.

## Materials and methods

### Patients

Membrane-bound Hsp70 (mHsp70) expression was assessed using inverted live-cell confocal microscopy in intraoperative biopsy specimens collected from newly diagnosed, treatment-naïve neuro-oncology patients (n = 23) between September 2020 and April 2026 (**Table 1**). The study was designed and carried out in compliance with the principles of the Declaration of Helsinki and received approval from the Ethics Committee of the Almazov Medical Research Centre (approval No. 2712-20, August 21, 2020). Written informed consent was obtained from all participants prior to inclusion. All experimental procedures adhered strictly to applicable regulatory standards and institutional guidelines.

**Table 1:** Demographic, clinical, and histopathological characteristics of the pediatric and adult study cohorts.

Pediatric patients	
Variables	Number (%)
Number of patients	20 (25%)
Age (years)	7.7 (range 2 – 16)
Lansky scale before surgery	70 (range 50 – 90)
Dexamethasone before surgery (mg)	4.5 (range 2 – 8)
Histology	
Glioblastoma IDH-wildtype, grade 4	13 (65%)
Atypical teratoid rhabdoid tumor, grade 4	7 (35%)
Adult patients	
Variables	Number (%)
Number of patients	60 (75%)
Age (years)	56.5 (range 42 – 69)
Karnofsky performance scale before surgery	70 (range 60 – 70)
Dexamethasone before surgery (mg)	12 (range 12 – 16)
Histology	
Glioblastoma, IDH-wildtype, grade 4	20 (33%)
Oligodendroglioma, IDH-mutant and 1p/19q-codeleted, grade 2	40 (67%)

Eligible participants were selected based on radiological findings consistent with malignant glioma, characterized on MRI by ring-enhancing lesions with irregular, thickened margins and a central region indicative of necrosis. Patients were excluded if imaging demonstrated tumor involvement of midline structures, the basal ganglia, or the brainstem.

### Magnetic resonance imaging

All MRI examinations were performed using a 3.0 Tesla whole-body MRI scanner (MAGNETOM Trio A Tim 3T, Siemens Healthineers). A standardized neuro-oncological imaging protocol was applied for all participants to ensure consistency of image acquisition and comparability across cases.

Signal acquisition was performed using a dedicated multi-channel head coil to optimize image quality and signal-to-noise ratio. The routine conventional MRI protocol included multiplanar structural sequences for anatomical assessment and lesion characterization. Pre-contrast T2-weighted fast spin-echo images were obtained in axial, coronal, and sagittal planes to evaluate tumor morphology, surrounding edema, and mass effect. Axial T2-weighted fluid-attenuated inversion recovery (FLAIR) imaging was also acquired to improve visualization of peritumoral hyperintensity and non-enhancing infiltrative abnormalities. T1-weighted images were obtained before and after intravenous gadolinium-based contrast administration to assess lesion enhancement, necrosis, and blood-brain barrier disruption. High-resolution three-dimensional post-contrast T1-weighted volumetric imaging was additionally performed to facilitate accurate tumor segmentation, surgical planning, and subsequent quantitative image analysis.

### Study design

A retrospective analysis was performed on preoperative MRI data obtained from patients with intracerebral gliomas. The study consisted of two partially overlapping cohorts: a radiomics cohort used for machine-learning model development and a biologically validated subgroup used for correlation with membrane-bound Hsp70 (mHsp70) expression.

The primary radiomics cohort included 80 patients. This cohort comprised 20 adult patients with glioblastoma (GBM), 40 adult patients with lower-grade glioma (LGG), and 20 pediatric patients with GBM/ATRT. The LGG cohort was included as a biologically and radiologically relevant comparison group, representing tumors with lower invasive potential and lower expression of invasion-associated biomarkers. This strategy was adopted because spatially matched histopathological ground truth for invasion is not available in clinical practice and because non-neoplastic peritumoral tissue suitable for comparison was not available.

**Figure 1:**
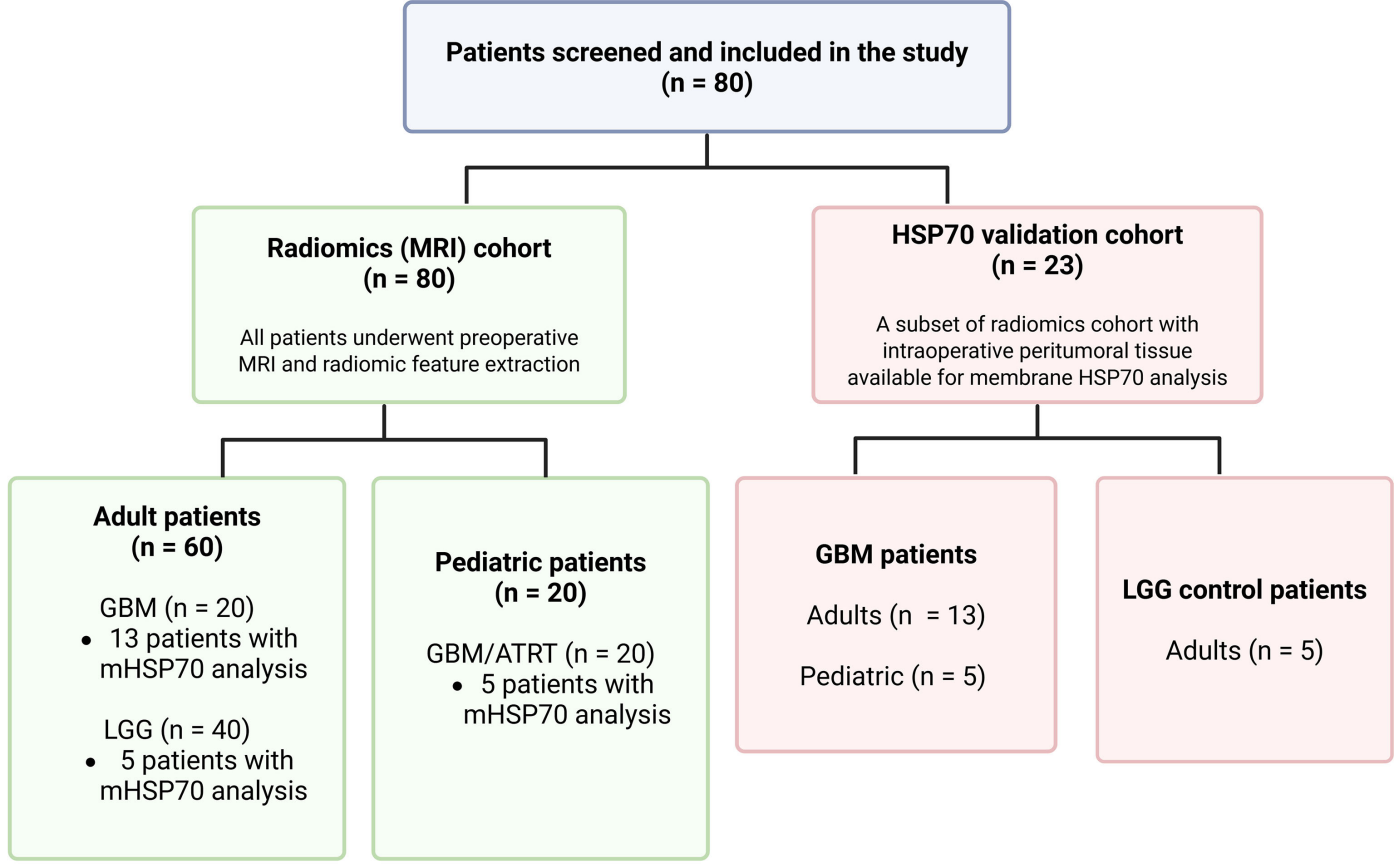
Flowchart of patient inclusion and allocation to the radiomics and mHsp70 validation cohorts.

**Figure 2:**
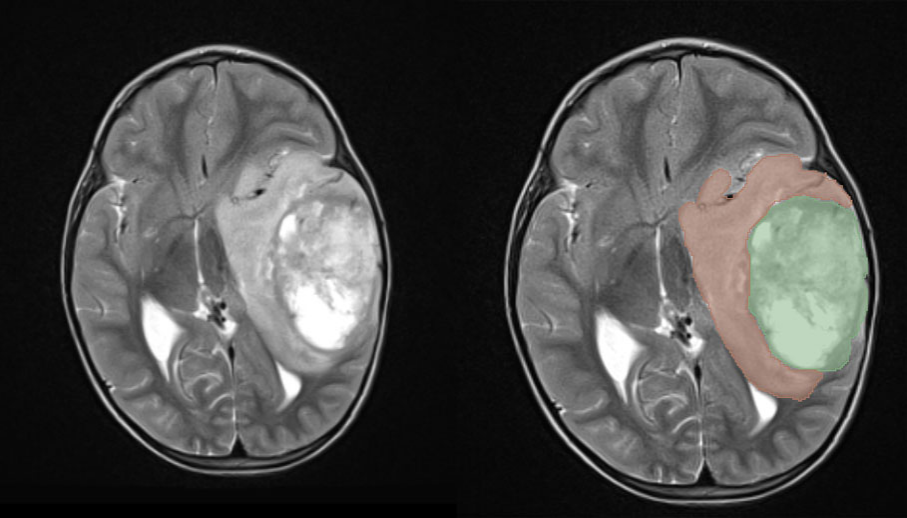
Contrast-enhanced MRI, T2 mode. Segmentation of the tumor of the left temporal lobe, the zone of the contrast-accumulating tumor core is highlighted in green, the zone of perifocal edema is highlighted in red. (Atypical teratoid rhabdoid tumor, GIV).

A subset of 23 patients from the radiomics cohort additionally underwent confocal microscopy analysis of membrane-bound Hsp70 expression in peritumoral tissue samples. This biologically validated subgroup included 18 patients with GBM (13 adults and 5 pediatric patients) and 5 adult LGG control patients. For these patients, patient-level Invasion Burden Index (IBI) values derived from radiomic invasion maps were correlated with mHSP70 fluorescence intensity (**Figure 1**). Patients were excluded if they had undergone previous surgical intervention, chemotherapy, or radiotherapy prior to MRI acquisition. Tumor entities other than glioma, including ependymoma and choroid plexus tumors, were also excluded. All patients underwent brain MRI including T2-weighted imaging. MRI studies were manually segmented using 3D Slicer software. The contrast-enhancing tumor core and the surrounding peritumoral edema zone were delineated and used for subsequent voxel-wise radiomic feature extraction and machine-learning analysis (**Figure 2**). Inter-rater segmentation reproducibility was assessed in five independently segmented MRI cases. The mean Dice similarity coefficient was 0.89 (range: 0.84–0.93), demonstrating good agreement between observers.

### Radiomic feature extraction

Extraction of radiomics features of selected segments was performed using the Pyradiomics software (30). Three image transformation categories were used: original images, wavelet-filtered images, and Laplacian of Gaussian (LoG) filtered images with σ = 1.0, 3.0, and 5.0. Local binary pattern analysis in three dimensions (LBP3D) was performed with a bin width of 1.0. Texture and intensity features were extracted from first-order statistics, gray-level co-occurrence matrix (GLCM), gray-level run-length matrix (GLRLM), gray-level size-zone matrix (GLSZM), and gray-level dependence matrix (GLDM). For GLCM features, a comprehensive subset including autocorrelation, joint average, cluster-based measures, contrast, correlation, entropy-related indices, and inverse difference measures was selected. Image preprocessing included intensity normalization (scale = 100), resampling to isotropic voxel spacing of 1×1×1 mm³ using B-spline interpolation, and gray-level discretization with a bin width of 25. Mask correction was enabled, and voxel intensity shifting (voxelArrayShift = 300) was applied to stabilize texture computation. Voxel-wise feature extraction was performed using a kernel-based neighborhood approach with a kernel radius of 1 voxel and masked kernel computation. A kernel radius of 1 voxel was selected to preserve spatial resolution during voxel-wise radiomic mapping of the peritumoral zone. Combined with the force2D setting, this enabled computation of localized texture descriptors while minimizing spatial smoothing of small-scale imaging heterogeneity. Features were calculated in batches of 10,000 voxels to optimize memory utilization. Although two-dimensional feature forcing was activated, features were ultimately derived from volumetric image data. Feature extraction was performed in accordance with the Image Biomarker Standardisation Initiative (IBSI) recommendations.

Additional image transformations included three-dimensional Local Binary Pattern (LBP3D) filtering implemented in PyRadiomics v3.0.1 using default settings with a bin width of 1.0. LBP3D-derived features were evaluated as supplementary texture descriptors but are not part of the IBSI reference feature set.

To assess feature reproducibility, five cases were independently re-segmented. Because the proposed framework operates on voxel-wise radiomic feature maps, reproducibility was evaluated by matching voxels between segmentations using their original flattened spatial coordinates and calculating ICC (2,1) for the 20 features with the highest mean absolute SHAP values.

### Machine-learning model

To computationally characterize imaging signatures of tumor infiltration, a supervised machine-learning framework based on a linear support vector machine (SVM) optimized using stochastic gradient descent (SGD) was implemented. The linear SVM classifier was selected because of its computational efficiency and suitability for high-dimensional radiomic datasets containing large numbers of voxel-wise features. Compared with more computationally intensive nonlinear models, linear SVMs trained with SGD provide scalable optimization, reduced memory requirements, and improved robustness when handling large voxel-level datasets.

The model learns a linear decision function defined as:


$$f(x)=\sum_{i=1}^{n}w_{i}x_{i}+b$$

where *x_i_* represents individual radiomic feature values, *w_i_* denotes the learned feature weights assigned by the classifier, *b* is the bias term, and *f(x)* corresponds to the voxel-wise decision score describing the relative likelihood of invasive tumor behavior.

The classifier was trained to discriminate between invasive and non-invasive tissue patterns using high-dimensional radiomic features extracted from conventional MRI sequences. This approach enables efficient handling of large feature spaces and supports scalable learning across millions of voxels while maintaining robustness through regularization.

Model training was performed using the hinge loss function, corresponding to a linear SVM formulation:


L=max(0,1−y·f(x))

where *L* denotes the hinge loss function, *y* represents the class label (-1 for non-invasive tissue and +1 for invasive tissue), and *f(x)* is the classifier decision function. This formulation penalizes misclassified samples while maximizing the separation margin between invasive and non-invasive classes.

A linear support vector machine optimized using stochastic gradient descent (SGD) was selected because SGD provides computationally efficient optimization for large-scale, high-dimensional datasets such as voxel-wise radiomic feature matrices. Compared with conventional quadratic optimization approaches for SVM training, SGD reduces computational burden and memory usage while allowing effective regularization and scalable learning across millions of voxel-level observations. Voxel-wise radiomic features were extracted from conventional MRI sequences following image preprocessing and intensity normalization. Prior to model training, all radiomic features were standardized to zero mean and unit variance using z-score normalization to ensure comparability across patients and imaging modalities. To reduce feature redundancy and improve model stability, low-variance features were removed using a variance threshold filtering approach. Features with near-constant variance across voxels were excluded prior to classification, reducing noise and mitigating overfitting in the high-dimensional feature space. The classifier was trained using labeled voxel-wise data derived from biologically defined tumor compartments, enabling the model to learn associations between imaging-derived radiomic patterns and invasive tumor behavior.

After fixed per-patient voxel subsampling, the training dataset contained 205,000 LGG-associated voxels and 200,000 GBM-associated voxels, resulting in a nearly balanced voxel-level class distribution. In addition, balanced class weighting was applied during model training to further mitigate residual imbalance.

To prevent information leakage, dataset partitioning was performed at the patient level prior to model training. All voxels originating from a given patient were assigned exclusively to a single dataset partition, ensuring that no patient contributed data to more than one cohort. The radiomics cohort (*n* = 80 patients) was divided into a training cohort (*n* = 50 patients), a validation cohort (*n* = 13 patients), and an independent test cohort (*n* = 17 patients). The resulting voxel-wise feature matrices were used for model development, validation, and final performance evaluation.

Radiomic features were extracted from preprocessed MRI data for all patients. Feature scaling was performed after dataset partitioning using parameters derived from the training cohort and subsequently applied to the validation and test cohorts. The validation cohort was used for model selection and optimization, whereas the independent test cohort remained completely held out until final model evaluation.

The trained classifier generated a continuous voxel-wise decision score corresponding to the signed distance from the separating hyperplane. Positive values indicated invasion-like radiomic signatures, whereas negative values reflected non-invasive tissue characteristics. This continuous scoring strategy preserved spatial gradients of tumor infiltration and enabled assessment of intratumoral heterogeneity beyond binary classification. For each patient, voxel-level predictions were aggregated to generate three-dimensional spatial invasion maps, providing a visual representation of predicted invasive activity within the tumor and surrounding peritumoral edema zone. In addition to spatial mapping, several patient-level summary metrics were derived from the voxel-wise score distribution, including the median invasion score, maximum invasion score, and hotspot-based metrics calculated from the upper decile (top 10%) of voxel scores. These hotspot-derived measures were designed to emphasize focal regions of pronounced invasion potentially associated with aggressive tumor behavior and treatment resistance. Together, this framework integrates voxel-wise radiomic analysis with machine-learning-based inference to provide both localized and global characterization of glioma invasiveness, supporting downstream biological correlation with mHsp70 expression.

### Histological analysis

Biopsy specimens were systematically collected from three spatially distinct tumor compartments, defined through preoperative MRI-based planning: the central necrotic core, identified as a non-enhancing region on T1-weighted images; the contrast-enhancing tumor portion visible on post-contrast T1-weighted sequences; and the peritumoral zone located approximately 5 mm beyond the enhancing margin, corresponding to hyperintense areas on FLAIR images (as described in (22, 31)). This spatially resolved sampling strategy was designed to capture intratumoral heterogeneity and the transition toward infiltrative tumor growth. Histopathological classification and grading were performed in accordance with the criteria established in the fifth edition of the World Health Organization classification of central nervous system tumors (2021). Following surgical retrieval, all specimens underwent an initial gross examination. Tissue samples without evident macroscopic necrosis were fixed in 10% neutral buffered formalin for 24 hours at room temperature (18–20 °C). Subsequently, the specimens were rinsed in distilled water for approximately 2 hours and processed through a graded series of alcohol solutions for dehydration before being embedded in paraffin. Paraffin blocks were sectioned using a microtome to obtain slices with a thickness of approximately 4–6 μm. These sections were mounted onto glass slides pretreated with heated alcohol to enhance adhesion. Deparaffinization was performed using xylene for 1–2 minutes, followed by rehydration through immersion in 96% ethanol and subsequent rinsing in distilled water. Standard hematoxylin and eosin (H&E) staining was then carried out, with hematoxylin applied for 5–10 minutes and eosin for approximately 1 minute. After staining, sections were again passed through ethanol for differentiation and washed in distilled water. The slides were then dried, cleared in xylene, and permanently mounted under coverslips using Canada balsam. Formalin-fixed, paraffin-embedded (FFPE) tissue sections were subsequently subjected to immunohistochemical analysis using an automated staining platform (Lab Vision Autostainer 360, Thermo Fisher Scientific). A panel of primary antibodies was applied to characterize tumor phenotype and molecular features, including GFAP (polyclonal; DakoCytomation, Denmark), synaptophysin (clone 27G12; DakoCytomation, Denmark), Olig2 (clone OLIG2; Abcam), Ki-67 (clone MIB1; DakoCytomation, Denmark), IDH1 R132H (clone H09; Dianova, Germany), MGMT (clone MT3.1; Diagnostic BioSystems, USA), ATRX (clone D5; Diagnostic BioSystems, USA), and EGFR (clone 31G7; Diagnostic BioSystems, USA). In cases demonstrating oligodendroglial histological features, the status of chromosomal arms 1p and 19q was further evaluated using fluorescence *in situ* hybridization (FISH), enabling molecular confirmation of tumor subtype.

### Live confocal microscopy of tumor specimens

During surgery, small tissue fragments (approximately 50–100 mm³) were collected and immediately processed. Samples were rinsed three times in phosphate-buffered saline (PBS) at room temperature with gentle agitation and subsequently incubated for 1 hour in a staining solution containing TMRM (1 μmol/L), Hoechst 33342 (0.1 μg/mL), and FITC-conjugated anti-Hsp70 monoclonal antibodies (SPA-810, StressMarq). These markers enabled visualization of mitochondrial membrane potential, nuclear DNA, and membrane-bound mHsp70, respectively.

Following incubation, specimens were washed with PBS and transferred to glass-bottom dishes (Ibidi μ-Dish, 35 mm), then covered with a coverslip to ensure stable positioning. Imaging was performed using an inverted confocal laser scanning microscope (Leica TCS SP8, Leica Microsystems) equipped with argon and helium-neon lasers. Fluorescence signals were acquired sequentially across three channels: Hoechst 33342 (excitation 405 nm, emission 415–500 nm), FITC-labeled Hsp70 (excitation 488 nm, emission 495–560 nm), and TMRM (excitation 561 nm, emission 565–700 nm). Imaging was conducted using a 63× oil immersion objective (HC PL APO 63×/1.40), with standardized acquisition settings maintained throughout all experiments. Images were recorded at a resolution of 1024 × 1024 pixels with line averaging (*n* = 3). Unstained samples served as controls to account for autofluorescence. For each specimen, at least 10 fields of view were analyzed. Quantification of Hsp70 signal intensity was performed after background subtraction using ImageJ (rolling ball radius: 50 pixels). Processed images were further analyzed in R using the EBImage package to extract mean fluorescence intensities of the green channel.

### Statistical analysis

Statistical analyses were performed in Python (version 3.9.21) using the SciPy, NumPy, Pandas, and statsmodels libraries, with data visualization generated using Matplotlib and Seaborn. Continuous variables were summarized as median and interquartile range (IQR) or mean ± standard deviation (SD), as appropriate based on distribution characteristics. Because of the limited sample size and non-Gaussian distribution of several variables, non-parametric statistical methods were applied. Differences between independent groups (e.g., low-grade glioma versus glioblastoma cohorts) were assessed using the two-sided Mann-Whitney U test. Associations between patient-level radiomic invasion metrics, including the Invasion Burden Index (IBI), and mHsp70 fluorescence intensity were evaluated using the Spearman rank correlation coefficient (ρ), which measures monotonic relationships without assuming normality. Correlation strength was interpreted descriptively according to the absolute value of ρ. All statistical tests were two-sided, and a p-value < 0.05 was considered statistically significant. Where relevant, exact p-values and corresponding effect size estimates were reported.

## Results

### Membrane-bound Hsp70 is selectively overexpressed in brain tumors

**Figure 3:**
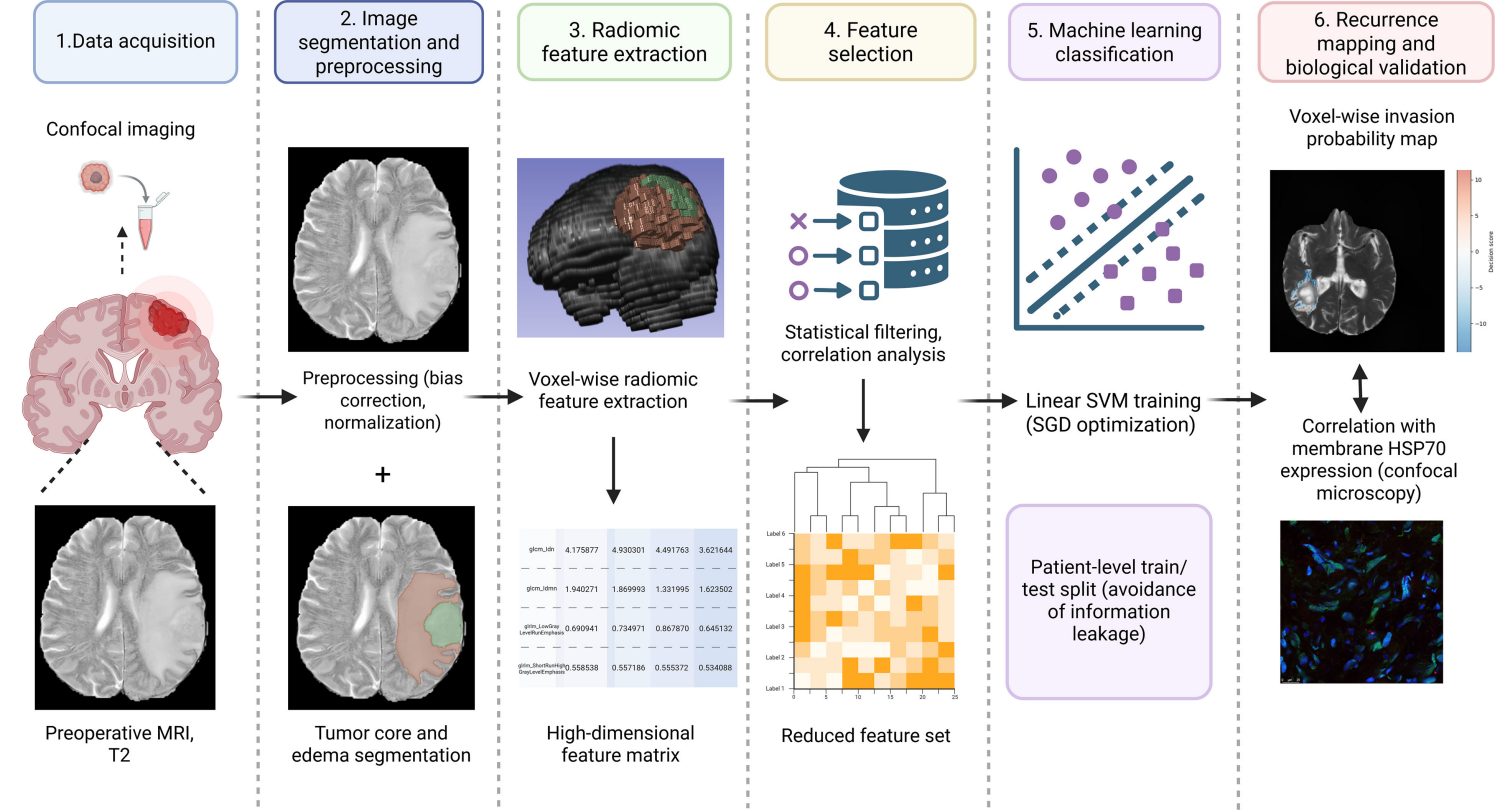
Schematic overview of the study design and analytical workflow for radiomic invasion mapping and biological validation.

The expression of membrane-bound Hsp70 (mHsp70) was assessed using live-cell inverted confocal microscopy on intraoperative biopsy specimens from adult and pediatric (*n* = 23) patients with primary brain tumors. The overall workflow for tumor sample preparation and analysis is outlined in **Figure 3**. Tumor tissues were collected from three spatially defined regions determined preoperatively via MRI, following the approach described by Hubert et al. (31): the tumor core, appearing hypointense or non-enhancing on T1-weighted images, corresponding to the necrotic center; the hyperintense region on FLAIR imaging; and the peritumoral zone, located approximately 5 mm beyond the enhancing margin on FLAIR sequences. Cell viability within the collected specimens was verified using TMRM staining, which highlights metabolically active mitochondria. In addition, tumor samples were co-stained with fluorophore-labeled 5-aminolevulinic acid (5-ALA), a compound used intraoperatively to delineate contrast-enhancing tumor tissue due to its selective accumulation as fluorescent porphyrins in malignant gliomas. Analysis revealed pronounced heterogeneity in both the localization and intensity of mHsp70 expression across all three regions. Representative confocal images from glioblastoma patients show strong mHsp70 immunoreactivity, particularly within the contrast-enhancing and peritumoral areas identified by MRI. This pattern of expression confirms that mHsp70 is upregulated in tumor cells while remaining largely absent in surrounding normal brain tissue, supporting its potential as a biomarker of invasive tumor populations.

Tumor tissue obtained during surgical resection was processed for live confocal microscopy to assess mHsp70 expression and related cellular fluorescence markers. In parallel, preoperative MRI scans underwent tumor segmentation and image preprocessing, followed by voxel-wise radiomic feature extraction from tumor and peritumoral regions. Extracted features were ranked according to importance using machine learning interpretability methods, and the most informative radiomic variables were selected for downstream analysis. Correlation structure among selected features was evaluated using hierarchical clustering and clustered heatmap visualization. Selected radiomic features and confocal microscopy-derived biological measurements were subsequently integrated into a support vector machine (SVM) machine learning classifier to generate voxel-wise invasion predictions. Model outputs were visualized as spatial peritumoral invasion maps, highlighting regions with higher predicted invasion-associated signal beyond the conventional MRI-defined tumor margin. This multimodal workflow enabled linkage of quantitative imaging phenotypes with mHsp70 expression and invasive tumor biology.

### Radiomic signatures of glioma invasion linked to mHsp70 in the peritumoral zone

**Figure 4:**
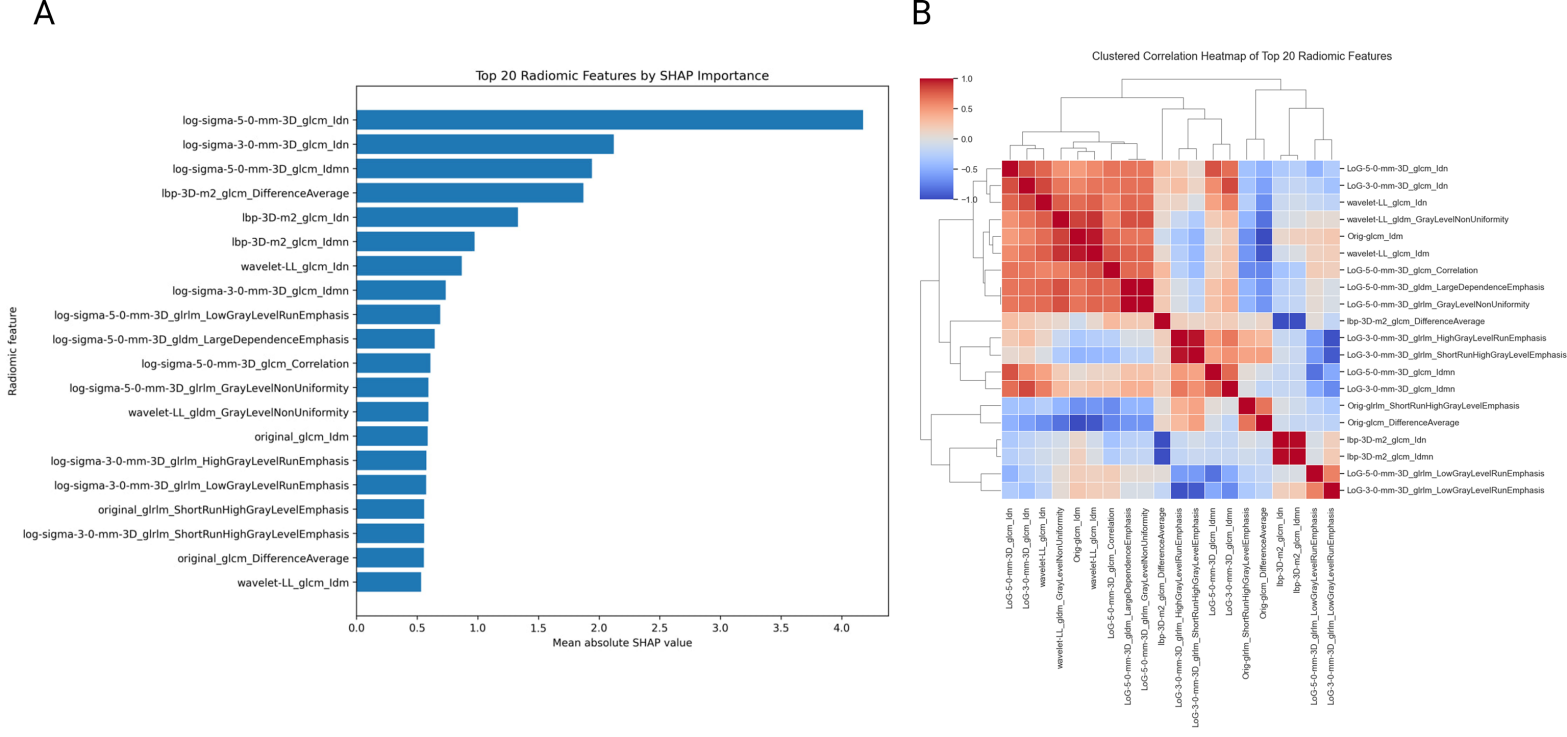
SHAP-based identification and correlation structure of the most informative radiomic features associated with glioma invasion. **(A)** Top 20 radiomic features ranked by SHAP importance. Bar plot showing the mean absolute SHAP values for the most influential radiomic features in the linear support vector machine (SVM) classifier optimized using stochastic gradient descent (SGD). Features derived from Laplacian-of-Gaussian (LoG) filtered images at sigma levels of 3–5 mm dominate the ranking, followed by texture features from GLCM, GLRLM, and GLDM matrices. **(B)** Hierarchically clustered Spearman correlation heatmap of the top 20 SHAP-selected radiomic features. Red indicates positive correlation and blue indicates negative correlation. Several coherent clusters were observed among GLCM and GLRLM texture metrics, demonstrating that the radiomic signature captures multiple related but non-identical aspects of peritumoral tissue heterogeneity. The data present a hierarchically clustered Spearman correlation heatmap of the top 20 radiomic features selected by SHAP importance analysis. Several coherent feature clusters were identified, indicating that multiple selected predictors capture related imaging characteristics. A prominent positively correlated cluster was observed among multiple GLCM-derived texture features, particularly LoG- and wavelet-filtered measures such as Idn, Idmn, Correlation, and Inverse Difference Moment. These metrics reflect local gray-level homogeneity and spatial dependence, suggesting that texture smoothness and structural regularity contribute substantially to model discrimination. A second cluster consisted primarily of GLRLM features, including GrayLevelNonUniformity, ShortRunHighGrayLevelEmphasis, and LowGrayLevelRunEmphasis. These variables characterize run-length heterogeneity and intensity distribution patterns, implying that intratumoral microstructural complexity is relevant to invasion status. Negative correlations were also observed between some GLCM homogeneity metrics and GLRLM heterogeneity measures, indicating that the radiomic signature contains features describing complementary rather than redundant imaging phenotypes. Overall, the clustering pattern suggests that the model’s top-ranked predictors represent several biologically meaningful and partially independent texture domains, rather than a single duplicated signal.

Radiomic feature importance was assessed using SHAP (SHapley Additive exPlanations) analysis of the trained SVM model. The top 20 features ranked by mean absolute SHAP value are presented in **Figure 4**. The most influential features were predominantly derived from Laplacian-of-Gaussian (LoG) filtered images at spatial scales of 3–5 mm, as well as higher-order texture features, particularly those based on gray-level co-occurrence matrix (GLCM) metrics such as inverse difference normalized and inverse difference moment normalized.

Additional contributions were observed from local binary pattern (LBP) features and wavelet-transformed texture descriptors, indicating that both local microtexture and multi-scale heterogeneity play a role in model decision-making. The prominence of GLCM, GLRLM, and GLDM features suggests that spatial intensity relationships and structural heterogeneity are key determinants of the radiomic invasion signature.

These findings indicate that the model captures complex textural patterns associated with tumor infiltration rather than relying solely on intensity-based features.

**Figure 5:**
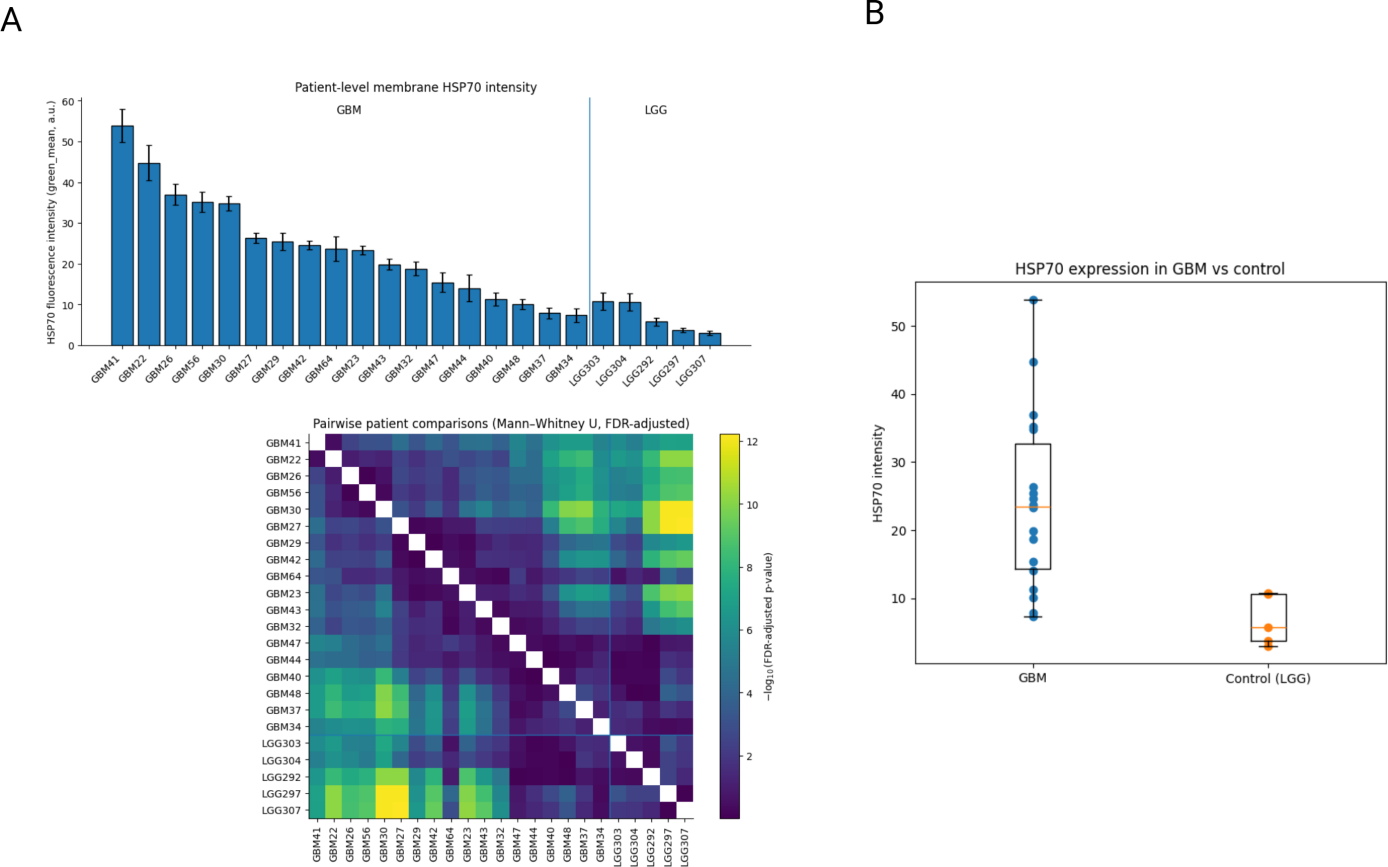
HSP70 fluorescence intensity in high grade and control groups. **(A)** Pairwise patient comparison heatmap of mHsp70 fluorescence intensity. Each cell represents the statistical difference in mHsp70 intensity between two patients based on Mann-Whitney U testing with Benjamini-Hochberg correction. Color intensity corresponds to -log10(adjusted p-value), with brighter colors indicating stronger statistical separation. Cross-cohort comparisons between glioblastoma (GBM) and low-grade glioma (LGG) patients generally showed stronger differences than within-cohort comparisons, supporting grade-associated variation in mHsp70 expression. **(B)** Boxplot comparing fluorescence intensity of mHsp70 between GBM and control (LGG) groups. Individual data points are overlaid. The high-grade tumors group demonstrates significantly higher mHsp70 expression compared to controls (Mann-Whitney U test, p = 0.0017), supporting its relevance as a biological marker.

To further explore the biological relevance of the radiomic invasion signature, confocal imaging-derived mHsp70 expression was analyzed and compared between tumor and control groups (**Figure 5**). Pairwise comparison heatmap analysis demonstrated broad differences in mHsp70 fluorescence intensity between glioblastoma and low-grade glioma patients (**Figure 5A**). Cross-cohort comparisons frequently showed stronger statistical separation than within-cohort comparisons, indicating distinct mHsp70 expression profiles between tumor grades. In contrast, comparisons within the GBM and LGG cohorts were generally weaker, although several GBM patients exhibited markedly elevated mHsp70 intensity relative to other GBM cases, reflecting biological heterogeneity.

A linear support vector machine (SVM) optimized using stochastic gradient descent (SGD) was trained on voxel-wise radiomic features extracted from the peritumoral region. Following variance-based feature filtering and feature standardization, the model demonstrated stable performance across cross-validation training, validation, and independent test cohorts.

**Figure 6:**
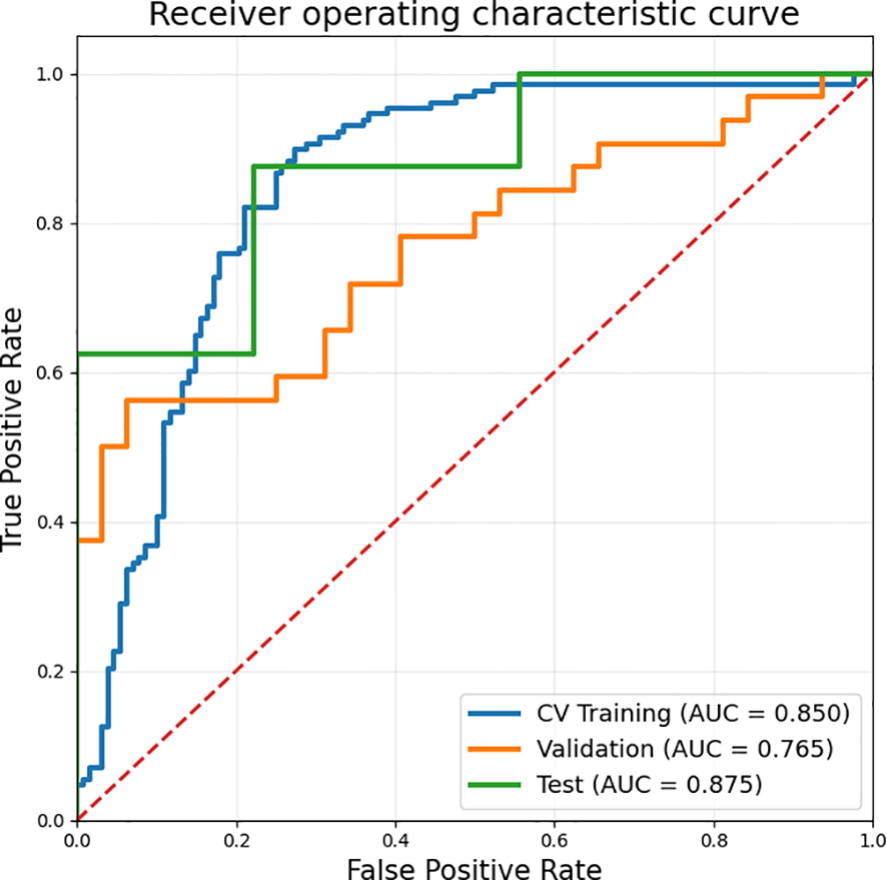
Receiver operating characteristic (ROC) analysis of the linear support vector machine (SVM) classifier optimized using stochastic gradient descent (SGD) for patient-level invasion classification based on voxel-wise radiomic features. The blue curve represents cross-validation training performance, demonstrating stable internal model discrimination with an area under the curve (AUC) of 0.850. The green curve represents validation performance, yielding an AUC of 0.765 and indicating consistent model generalization across validation folds. The orange curve represents independent test cohort performance, with a patient-level AUC of 0.875, demonstrating good discriminative ability for distinguishing invasive from non-invasive tumor phenotypes. The dashed diagonal line indicates the line of no discrimination (AUC = 0.50).

**Figure 7:**
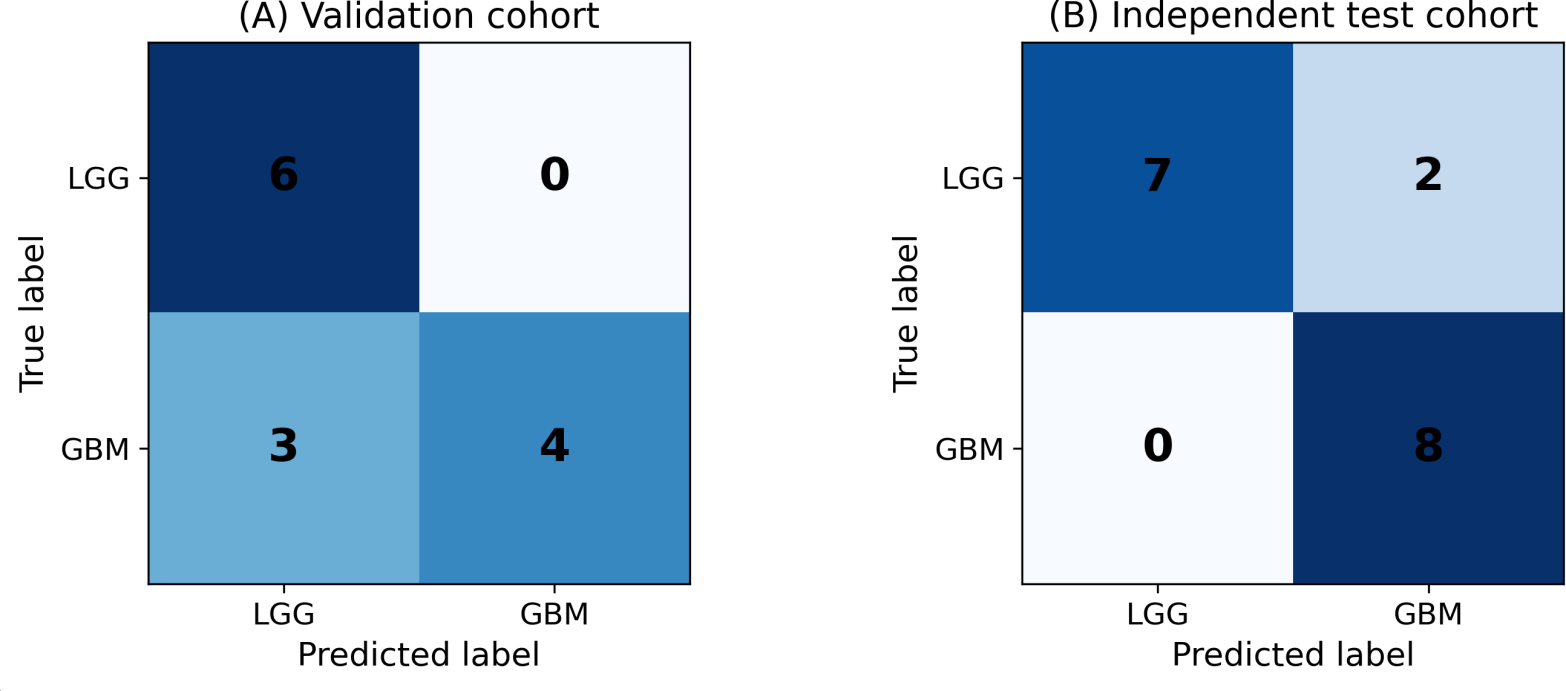
Confusion matrices for patient-level radiomic phenotype classification using the linear SVM model. **(A)** Validation cohort confusion matrix (accuracy 76.9%, sensitivity 57.1%, specificity 100%). **(B)** Independent test cohort confusion matrix (accuracy 88.2%, sensitivity 100%, specificity 77.8%).

In the training cohort, the model achieved an AUC of 0.850 (95% CI: 0.799–0.898). In the validation cohort, the model achieved an AUC of 0.765 (95% CI: 0.641–0.877). Independent test-set evaluation yielded an AUC of 0.875 (95% CI: 0.736–0.896), indicating good discriminative performance for distinguishing GBM-associated infiltrative radiomic phenotypes from lower-grade glioma phenotypes (**Figure 6**). Classification performance metrics are summarized in **Table 2**, and corresponding confusion matrices are shown in **Figure 7**.

**Table 2:** Classification performance of the linear support vector machine (SVM) model.

Cohort	AUC (95% CI)	Accuracy	Sensitivity	Specificity	Precision	F1-score
Training	0.850 (0.799–0.898)	0.812	0.898	0.727	0.767	0.827
Validation	0.765 (0.641–0.877)	0.769	0.571	1.000	1.000	0.727
Test	0.875 (0.736–0.896)	0.882	1.000	0.778	0.800	0.889

Voxel-wise classifier outputs were subsequently reconstructed into three-dimensional invasion maps, enabling spatial visualization of heterogeneous invasion-associated radiomic signatures within the peritumoral edema region.

### Invasion burden index

Voxel-wise inference using the SVM model generated spatial maps of invasion-associated radiomic scores within the segmented peritumoral zone. These maps revealed focal regions of elevated model-derived scores extending beyond the visually obvious lesion margin. To assess biological relevance, hotspot-based summary metrics were derived from the highest-scoring voxels within each patient.

Among the evaluated metrics, the Invasion Burden Index (IBI), defined as the mean score of the highest-scoring voxel subset, demonstrated the strongest association with membrane-bound mHsp70 expression measured by confocal microscopy.

**Figure 8:**
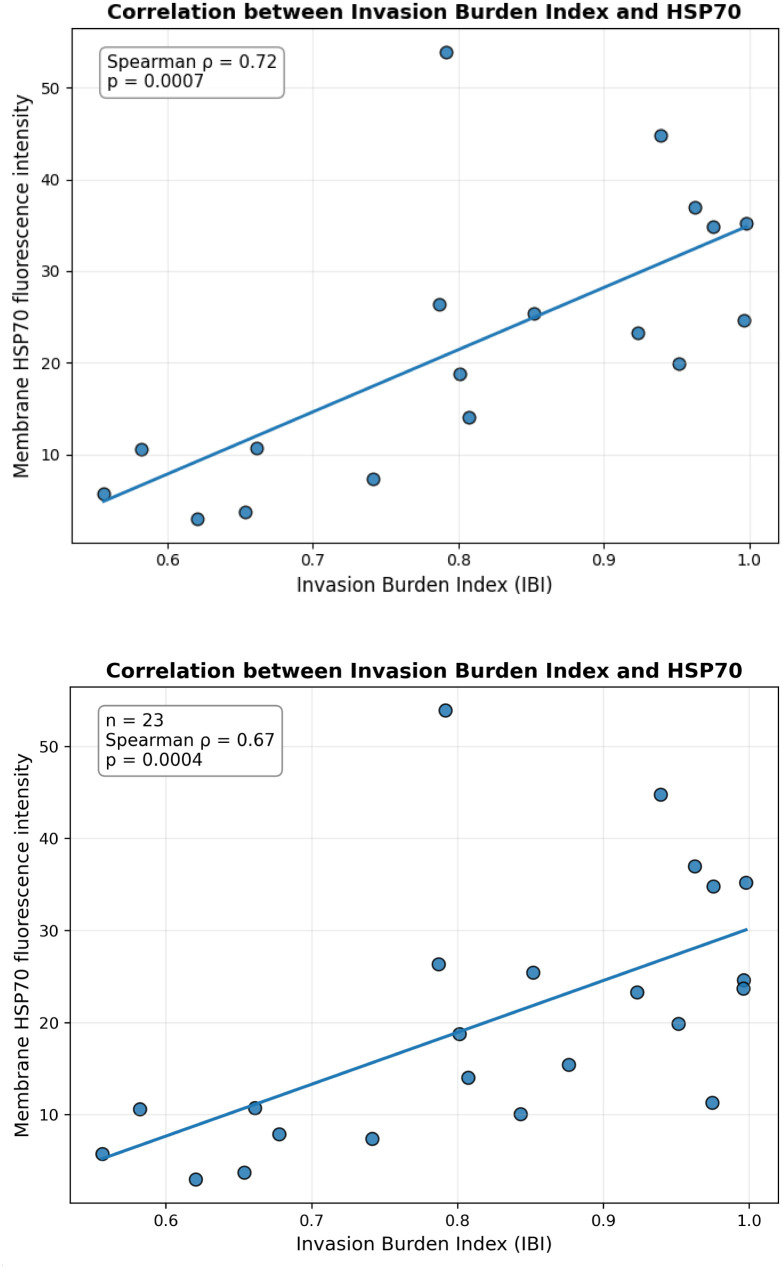
Association between the invasion burden index (IBI) and mHsp70 fluorescence intensity. Scatter plot demonstrating a positive relationship between patient-level IBI and mHsp70 fluorescence intensity (green channel) measured on confocal microscopy. Higher IBI values were associated with increased mHsp70 expression, consistent with a strong positive Spearman correlation (ρ = 0.67, p = 0.0004). The findings support IBI as a radiomics-derived marker of invasion-associated tumor biology.

**Figure 8** illustrates the association between the radiomics-derived invasion burden index (IBI) and mHsp70 fluorescence intensity measured on confocal microscopy. A clear positive trend was observed, with higher IBI values associated with increased mHsp70 expression. This visual pattern was supported by statistical analysis demonstrating a strong positive Spearman correlation between IBI and mean mHsp70 fluorescence intensity (green_mean_mean) (ρ = 0.67, p = 0.0004). Leave-one-out sensitivity analysis showed stable correlation coefficients across all iterations, indicating that the observed association was not driven by any individual patient. In contrast, the maximum voxel-wise invasion-associated radiomic score did not show a significant association with either fluorescence summary metric (median fluorescence: ρ = -0.08, p = 0.754; mean fluorescence: ρ = 0.03, p = 0.919). These findings indicate that the patient-level hotspot-based IBI is biologically more informative than isolated peak voxel values.

Using cohort-level stratification, patients were separated into low-IBI and high-IBI subgroups, representing distinct invasion-associated radiomic phenotypes. Higher IBI values were more frequently observed in glioblastoma patients, whereas lower values predominated in low-grade glioma controls. Taken together, these findings suggest that radiomic hotspot burden is associated with mHsp70 expression, a marker linked to aggressive tumor biology, and supports the biological relevance of the identified invasion-associated imaging phenotype.

**Figure 9:**
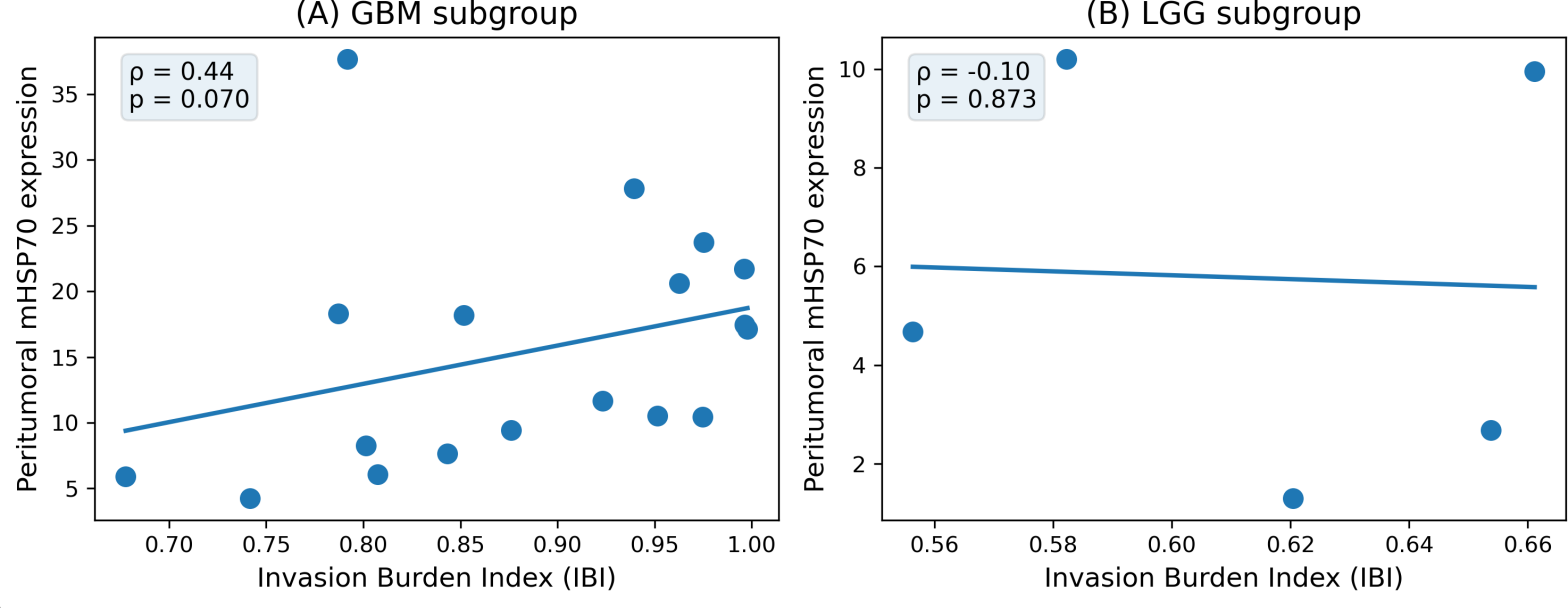
Stratified subgroup analysis of the relationship between Invasion Burden Index (IBI) and peritumoral mHsp70 expression. **(A)** GBM subgroup demonstrating a moderate positive association between radiomic infiltrative phenotype burden and peritumoral mHsp70 expression (Spearman ρ = 0.44, p = 0.07). Although statistical significance was not reached, the direction of the association was consistent with the overall cohort analysis. **(B)** LGG subgroup demonstrating no significant association between IBI and mHsp70 expression (Spearman ρ = -0.10, p = 0.87). Interpretation is limited by the small sample size of the LGG control cohort (n = 5).

Exploratory subgroup analyses were performed to assess potential confounding by tumor grade in the observed IBI-mHsp70 association. Within the GBM subgroup, a moderate positive association between radiomic infiltrative phenotype burden and peritumoral mHsp70 expression was observed (Spearman ρ = 0.44), although statistical significance was not reached (p = 0.07). Within the LGG subgroup, no significant association was identified (Spearman ρ = -0.10, p = 0.87); however, interpretation is limited by the small sample size of the control cohort (n = 5) (**Figure 9**).

These findings indicate that the overall IBI-mHsp70 association was accompanied by substantial biological heterogeneity within the GBM cohort. Although potential confounding by tumor grade cannot be excluded, the positive association trend observed within GBM suggests that the relationship may not be attributable solely to simple tumor-grade separation. Larger studies incorporating spatially co-registered molecular sampling will be required to further clarify the biological specificity of the radiomic infiltrative phenotype.

Because multiple radiomic summary metrics were evaluated, Benjamini-Hochberg false discovery rate correction was additionally performed. The association between IBI and mHsp70 expression remained significant after correction (FDR-adjusted p = 0.0013).

**Figure 10:**
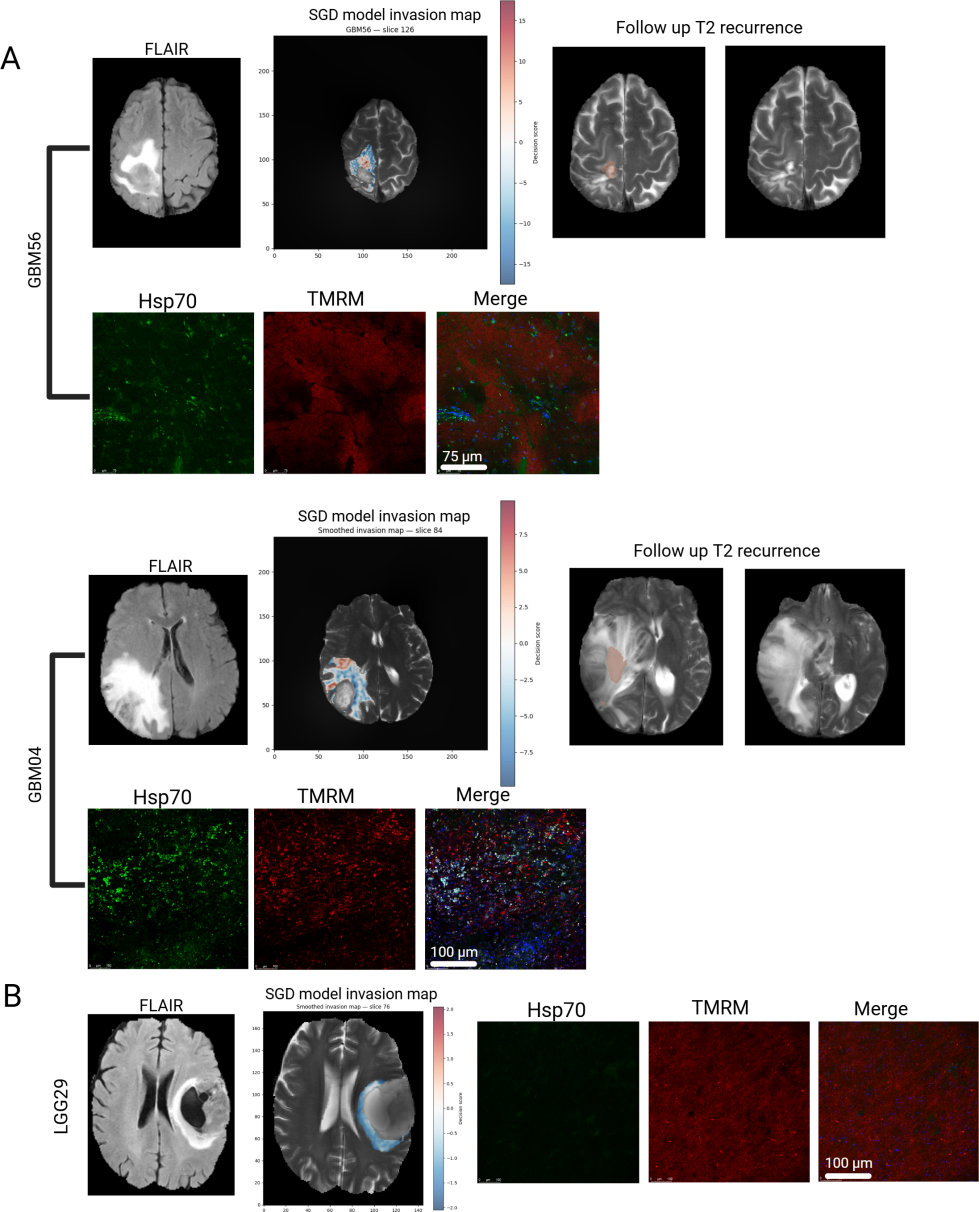
Representative multimodal comparison of a glioblastoma patients (GBM56, GBM04) and a low-grade glioma patient (LGG29) integrating MRI, voxel-wise invasion-associated radiomic score map, follow-up imaging, and confocal microscopy. **(A)** Preoperative FLAIR MRI demonstrates a high-grade lesion with extensive peritumoral signal abnormality. The corresponding voxel-wise invasion-associated radiomic score map generated from T2-weighted MRI shows focal high decision-score regions (warm colors), indicating radiomic hotspots associated with an invasion-like phenotype. On follow-up T2-weighted MRI, the site of tumor recurrence spatially corresponds to the previously predicted high-score hotspot region. Confocal microscopy from the same cases demonstrates strong mHsp70 fluorescence (green), mitochondrial membrane potential signal measured by TMRM (red), and merged-channel imaging, consistent with biologically active tumor tissue. **(B)** Preoperative FLAIR MRI demonstrates a low-grade glioma with comparatively circumscribed morphology. The corresponding invasion-associated radiomic score map shows uniformly low decision scores (cool colors) without focal hotspot regions. Confocal microscopy demonstrates low mHsp70 fluorescence intensity with corresponding TMRM and merged-channel images. The spatial comparison between radiomic score maps and follow-up imaging is intended as an illustrative example of the biological and imaging characteristics associated with the identified radiomic phenotype and should be interpreted as exploratory.

To further evaluate the spatial relevance of the radiomic signature, voxel-wise decision maps were generated and compared with follow-up imaging. Areas exhibiting elevated invasion-associated radiomic scores frequently demonstrated spatial correspondence with regions of subsequent tumor progression. Voxel-wise application of the trained SVM model enabled generation of spatial maps of invasion-associated radiomic scores within the peritumoral zone. An example is shown in **Figure 10**, where the SVM model decision score is visualized as a continuous heatmap overlaid on T2-weighted MRI. Regions of elevated invasion-associated radiomic score (red) are observed extending beyond the visually apparent tumor boundary into adjacent peritumoral tissue.

On follow-up imaging, some of these regions corresponded to sites of subsequent tumor progression, supporting the potential biological relevance of the identified radiomic phenotype. Given the limited number of available follow-up cases, this analysis should be considered exploratory.

Across cases, higher model-derived invasion scores were associated with increased mHsp70 expression, whereas lower scores were associated with lower mHsp70 signal and non-hotspot imaging phenotype. Warm colors indicate higher classifier decision scores; cool colors indicate lower scores. These maps represent a continuous model-derived radiomic scores reflecting similarity to the invasion-associated imaging phenotype identified during model training, rather than a calibrated probability of microscopic infiltration. The spatial distribution of high-score regions suggests that the model captures heterogeneity within the peritumoral environment that may be associated with infiltrative tumor biology. To explore the potential clinical relevance of the generated invasion-associated radiomic score maps, follow-up MRI data were analyzed. As illustrated in **Figure 10** the region of tumor recurrence identified on follow-up imaging demonstrated spatial correspondence with areas of elevated invasion-associated radiomic-associated radiomic score in the initial map. Notably, the direction of tumor progression appears to align with regions previously characterized by higher radiomic scores, suggesting that the identified imaging phenotype may reflect underlying biological processes associated with tumor spread. However, this observation is based on a limited number of cases and should be interpreted as exploratory. Further studies with larger cohorts and prospective validation will be required to determine whether these radiomic signatures are associated with future patterns of glioma progression.

## Discussion

Previous studies have demonstrated the potential of MRI-based approaches to characterize tumor biology, predict disease progression, and provide noninvasive biomarkers of treatment response (32, 33). In particular, advances in radiomics and machine learning have enabled the extraction of quantitative imaging features that capture subtle patterns of tumor heterogeneity beyond what can be appreciated by conventional radiological assessment (34). These approaches have shown promise for predicting molecular characteristics, clinical outcomes, and invasive growth patterns across a range of brain tumors. Thus, Cepeda et al. (35) utilized postoperative MRI to predict regions at risk of tumor recurrence. However, establishing direct biological correlates of imaging-derived signatures remains a major challenge, limiting the interpretability and translational applicability of many radiomic models. In contrast to prior work, the present study sought to investigate whether radiomic patterns within the peritumoral zone capture biologically meaningful signatures associated with glioma infiltration. To address this question, we combined voxel-wise radiomic analysis with biological validation using mHsp70. This approach is conceptually aligned with the hypothesis that invasion-related biological processes manifest as measurable alterations in radiomic texture patterns within the peritumoral zone. Therefore, the proposed model should be interpreted as detecting invasion-associated imaging phenotypes rather than predicting recurrence at a specific anatomical location within an individual patient.

A key finding of this study was the significant association between the radiomics-derived Invasion Burden Index (IBI) and mHsp70 expression in peritumoral tissue samples. Patients exhibiting higher radiomic hotspot burden demonstrated increased mHsp70 fluorescence intensity, suggesting that the identified radiomic phenotype reflects biologically relevant characteristics rather than imaging variation alone. The robustness of this relationship was supported by leave-one-out sensitivity analysis and by reproducibility analysis demonstrating good-to-excellent agreement for the most influential radiomic features.

Recent studies increasingly support the concept that radiomic signatures may reflect underlying tumor biology rather than serving only as imaging classifiers. Thus, Bernatowicz et al. demonstrated that a radiomic CT-TIME signature was associated with a T-cell inflamed tumor microenvironment and correlated with immune markers including CD3, CD8, and CD163, illustrating that imaging features can capture biologically meaningful microenvironmental states (36). Similarly, Noor et al. showed that MRI-derived radiomic features could predict transcriptomic high-risk groups in triple-negative breast cancer and stratify patients according to survival outcomes (37). These findings support the broader hypothesis that quantitative imaging features may act as non-invasive surrogates of molecular and cellular tumor phenotypes. A similar strategy was employed by Dasgupta et al., who trained a model using edema regions from patients with low-grade gliomas and brain metastases (10). In their design, peritumoral edema in metastases served as a surrogate for non-invasive tissue. The performance of our model exceeds that reported by Dasgupta et al., who achieved an AUC of 0.61 and an accuracy of 0.79. In contrast, the present study demonstrated a substantially higher AUC of 0.875, indicating improved discriminative ability of the model. In another study of Akbari et al., the authors reported a mean AUC of 0.84, with sensitivity and specificity of 91% and 93%, respectively, using a multiparametric imaging approach that incorporated structural MRI, diffusion tensor imaging, and perfusion imaging (11). Despite relying solely on radiomic features extracted from conventional MRI sequences, the present study achieved a comparable AUC of 0.875. While multiparametric imaging may improve sensitivity to tumor infiltration, such protocols are not routinely available in all clinical settings. In contrast, the use of standard MRI in the present study enhances the feasibility and potential for clinical translation of the proposed approach.

The relevance of peritumoral radiomics has also been demonstrated outside glioma invasion modeling. Xu et al. showed that combined intratumoral and peritumoral DCE-MRI radiomic signatures improved discrimination of luminal versus non-luminal breast cancer subtypes compared with intratumoral features alone (38). In glioblastoma, Chen et al. similarly reported that integrating intratumoral and peritumoral MRI-derived radiomic features improved prediction of MGMT promoter methylation status (39).

Importantly, this study also aimed to bridge imaging findings with underlying tumor biology. To our knowledge, this study is the first to combine machine learning-derived radiomic features with mHsp70 expression to generate an invasion burden index (IBI) capable of stratifying patients into low- and high-burden invasion phenotypes. This suggests that quantitative MRI may capture biologically meaningful information linked to mHsp70-associated tumor aggressiveness. The identification of high-IBI and low-IBI patient subgroups suggests that radiomic analysis may capture differences in invasion-associated imaging phenotypes within glioma. The association between elevated IBI and increased mHsp70 expression (Spearman ρ = 0.67, p = 0.0004) supports the hypothesis that the identified radiomic phenotype correspond to invasive tumor behavior. mHsp70 has been implicated in tumor aggressiveness (22), immune modulation (40), and treatment resistance (41, 42), thus providing biological context for the observed radiomic findings. Biological validation was performed using confocal microscopy of peritumoral tissue samples, where mHsp70 fluorescence intensity was quantified. However, it should be noted that confocal samples were not spatially co-registered with MRI-derived regions, and therefore the analysis reflects patient-level associations rather than voxel-wise correspondence. Future studies incorporating image-guided spatial sampling will be necessary to establish a direct relationship between local radiomic signatures and microscopic tissue characteristics.

Linking radiomic features with molecular markers such as IDH mutation status and MGMT promoter methylation has been extensively explored in recent radiogenomic studies, demonstrating that quantitative MRI-derived features can serve as non-invasive proxies of tumor biology (43–45). A growing body of literature shows that radiomic signatures are associated with key genetic and epigenetic alterations in gliomas, reflecting how molecular changes shape tumor microstructure and imaging appearance. Multiple studies have reported that IDH-mutant gliomas exhibit more homogeneous texture patterns, lower contrast enhancement, and less infiltrative margins on MRI, whereas IDH-wildtype tumors tend to present with increased heterogeneity, necrosis, and aggressive growth characteristics. Radiomic analyses capturing these features, particularly texture-based metrics such as gray-level co-occurrence matrix (GLCM) parameters and wavelet-derived features have shown strong predictive performance for IDH mutation status (45). Similarly, MGMT promoter methylation has been linked to distinct imaging phenotypes, including differences in edema, enhancement patterns, and intratumoral heterogeneity, which can also be quantified using radiomic approaches (46, 47). Recent glioma radiogenomic studies further demonstrate the ability of MRI radiomics to reflect molecular and prognostic tumor characteristics. Karakas et al. reported high diagnostic performance of radiomics-based machine learning models for IDH1 genotype prediction (34), while Tan et al. showed that MRI-derived radiomic features could predict EGFR expression and were associated with immune infiltration patterns in high-grade gliomas (48). Furthermore, Tixier et al. demonstrated that preoperative MRI radiomics improved survival stratification in glioblastoma beyond MGMT methylation status alone (49), while other research groups reported that combined clinical-radiomic models improved survival prediction in glioma cohorts (50–52). Together with studies linking radiomics to MGMT methylation and IDH status, reinforce the view that radiomic texture and intensity features may capture biologically meaningful variation in glioma microstructure.

The clinical importance of identifying invasion-prone tissue is further supported by surgical oncology data. The RANO resect group showed that lower residual contrast-enhancing tumor volume and more extensive resection of non-contrast-enhancing tumor were associated with improved survival in IDH-wildtype glioblastoma (53). This provides a clinical rationale for developing imaging tools capable of characterizing tumor infiltration beyond visible enhancing margins. In this context, voxel-wise radiomic mapping of invasion-associated phenotypes may eventually contribute to more individualized surgical planning, although prospective validation is required.

Several limitations should be acknowledged. First, the relatively small sample size limits statistical power and may reduce the generalizability of the findings to broader glioma populations. Second, although significant associations were observed between radiomic metrics and mHsp70 expression, the lack of precise spatial co-registration between MRI-derived hotspot regions and histological sampling sites may have attenuated or obscured stronger local imaging-biology relationships. Third, derivation of the patient-level invasion burdenindex (IBI) required aggregation of voxel-wise predictions into summary measures, which may mask fine-scale spatial heterogeneity and localized invasion patterns that are more directly reflected by high-value radiomic hotspot regions. Despite these promising findings, prospective validation is essential. In particular, the absence of longitudinal clinical follow-up data, including survival and recurrence outcomes, limited our ability to determine whether IBI has prognostic significance. Future prospective studies should therefore evaluate the relationship between IBI and overall survival, progression-free survival, recurrence pattern, and therapeutic response, as well as confirm its robustness across independent cohorts and imaging platforms. All patients included in this study received dexamethasone therapy, with doses ranging from 8 to 12 mg daily. Corticosteroids are known to reduce vasogenic edema (54) and alter the imaging characteristics of the peritumoral zone (55), which may influence radiomic feature extraction, particularly those related to texture, intensity, and spatial heterogeneity. As a result, steroid administration represents a potential confounding factor, as it may partially obscure or modify imaging signatures associated with tumor infiltration. However, because dexamethasone treatment was consistently administered across the entire cohort, this effect is likely systematic rather than random, thereby maintaining internal consistency of the dataset. Consequently, while steroid exposure may limit the direct biological interpretability of certain radiomic features and reduce generalizability to steroid-naïve populations, it also contributes to cohort homogeneity and reduces variability related to treatment status. Finally, reproducibility analysis was performed in a limited subset of five independently re-segmented cases. Although the most influential radiomic features demonstrated good-to-excellent agreement, larger multicenter studies will be necessary to confirm feature stability across different observers, institutions, MRI scanners, and acquisition protocols.

## Conclusion

This study demonstrates that voxel-wise radiomic analysis can move beyond conventional whole-tumor assessment to characterize spatial heterogeneity within the glioma peritumoral zone and identify imaging phenotypes associated with biologically relevant tumor characteristics. Using selected radiomic features, we derived a patient-level invasion burden index (IBI) that stratified patients into high- and low-invasion subgroups. Higher IBI values were strongly associated with increased mHsp70 fluorescence intensity, indicating that IBI may serve as a non-invasive biomarker of invasion-associated tumor biology.

A significant positive association was observed between IBI and mHsp70 expression, suggesting that the identified radiomic phenotype reflects biologically meaningful aspects of aggressive tumor behavior. Furthermore, SHAP-based interpretation and reproducibility analysis demonstrated that the most influential radiomic features were both biologically relevant and robust to segmentation variability. If validated in larger prospective studies, radiomic invasion mapping and the Invasion Burden Index (IBI) may have several potential clinical applications. These may include improved characterization of biologically aggressive glioma phenotypes, identification of spatially heterogeneous regions within the peritumoral environment, support for surgical and radiotherapy planning, and non-invasive monitoring of disease progression. Furthermore, the observed association between IBI and mHsp70 expression raises the possibility that radiomic analysis could contribute to the identification of tumors exhibiting biologically aggressive characteristics relevant to prognosis and biomarker-driven therapeutic strategies. At present, however, these potential applications remain investigational and require prospective validation with spatially matched biological sampling and longitudinal clinical outcomes before clinical implementation can be considered.

## Data Availability

The raw data supporting the conclusions of this article will be made available by the authors, without undue reservation.
